# Factors Associated with the Complications of Hysteroscopic Myomectomy

**DOI:** 10.1055/s-0040-1713915

**Published:** 2020-09-08

**Authors:** Mariana Patelli Juliani de Souza Lima, Lúcia Costa-Paiva, Luiz Gustavo Oliveira Brito, Luiz Francisco Baccaro

**Affiliations:** 1 Department of Obstetrics and Gynecology, Universidade Estadual de Campinas (UNICAMP), Campinas, SP, Brazil

**Keywords:** uterine myomectomy, leiomyoma, myoma, uterine hemorrhage, intraoperative complications, miomectomia uterina, leiomioma, mioma, hemorragia uterina, complicações intraoperatórias

## Abstract

**Objective** To evaluate the factors associated with complete myomectomy in a single surgical procedure and the aspects related to the early complications.

**Methods** A cross-sectional study with women with submucous myomas. The dependent variables were the complete myomectomy performed in a single hysteroscopic procedure, and the presence of early complications related to the procedure.

**Results** We identified 338 women who underwent hysteroscopic myomectomy. In 89.05% of the cases, there was a single fibroid to be treated. According to the classification of the International Federation of Gynecology and Obstetrics (Fédération Internationale de Gynécologie et d'Obstétrique, FIGO, in French), most fibroids were of grade 0 (66.96%), followed by grade 1 (20.54%), and grade 2 (12.50%). The myomectomies were complete in 63.31% of the cases, and the factors independently associated with complete myomectomy were the diameter of the largest fibroid (prevalence ratio [PR]: 0.97; 95% confidence interval [95%CI]: 0.96–0.98) and the classification 0 of the fibroid according to the FIGO (PR: 2.04; 95%CI: 1.18–3.52). We observed early complications in 13.01% of the hysteroscopic procedures (4.44% presented excessive bleeding during the procedure, 4.14%, uterine perforation, 2.66%, false route, 1.78%, fluid overload, 0.59%, exploratory laparotomy, and 0.3%, postoperative infection). The only independent factor associated with the occurrence of early complications was incomplete myomectomy (PR: 2.77; 95%CI: 1.43–5.38).

**Conclusions** Our results show that hysteroscopic myomectomy may result in up to 13% of complications, and the chance of complete resection is greater in small and completely intracavitary fibroids; women with larger fibroids and with a high degree of myometrial penetration have a greater chance of developing complications from hysteroscopic myomectomy.

## Resumo

**Objetivo** Avaliar os fatores associados a miomectomia por histeroscopia completa em um único procedimento e as suas complicações.

**Métodos** Estudo de corte transversal com mulheres submetidas a histeroscopia para exérese de miomas submucosos. As variáveis dependentes foram a miomectomia completa realizada em um tempo cirúrgico único, e a presença de complicações precoces relacionadas ao procedimento.

**Resultados** Analisamos 338 mulheres que foram submetidas a miomectomia histeroscópica. Em 89,05% dos casos, o mioma a ser tratado era único. Quanto à classificação da Federação Internacional de Ginecologia e Obstetrícia (Fédération Internationale de Gynécologie et d'Obstétrique, FIGO, em francês), a maioria era de grau 0 (66,96%), seguidos pelos graus 1 (20,54%) e 2 (12,50%). As miomectomias foram completas em 63,31% das mulheres, sendo que os fatores independentemente associados à miomectomia completa foram o diâmetro do maior mioma (razão de prevalência [RP]: 0,97; intervalo de confiança de 95% [IC95%]: 0,96–0,98) e a classificação FIGO grau 0 (RP: 2,04; IC95%: 1,18–3,52). Foram observadas complicações precoces em 13,01% dos procedimentos (4,44% apresentaram sangramento excessivo durante o procedimento, 4,14%, perfuração uterina, 2,66%, falso pertuito, 1,78%, intoxicação hídrica, 0,59%, laparotomia exploradora, e 0,3%, infecção pós-operatória). O único fator independentemente associado à ocorrência de complicações precoces foi a realização de miomectomia incompleta (RP: 2,77; IC95%: 1,43–5,38).

**Conclusão** Nossos resultados mostram que as complicações da miomectomia por histeroscopia podem ocorrer em até 13% dos procedimentos. A chance de ressecção completa é maior em miomas pequenos e completamente intracavitários; mulheres com miomas maiores e com maior grau de penetração miometrial têm maiores chances de desenvolver complicações.

## Introduction

Uterine fibroids are benign monoclonal tumors composed of smooth muscle cells mixed with different amounts of extracellular matrix, arising in the myometrium. 1 2 Fibroids are the most frequent benign tumors in women of reproductive age, and the prevalence rates vary between 20% and 50%. 3 4 Although almost always benign 5 and with a low rate of cell proliferation in vivo, they can lead to increased uterine bleeding, pelvic pain and infertility, 3 depending on their size and location within the uterus. Submucosal myomas extend into the uterine cavity and can cause the greatest changes in the integrity of the endometrium and in the capacity of the myometrium to contract and stop the bleeding from the endometrial vessels. 1

For women with submucosal myomas, myomectomy through hysteroscopy is an option that enables uterine preservation, 6 and it is currently considered the procedure of choice for the treatment of this disease. 7 Before the advent of hysteroscopy, many intrauterine diseases were treated with more invasive procedures, with greater risks and costs, such as laparotomy and hysterectomy, or less resolution, such as dilation and curettage. 8 However, among the procedures that can be performed by hysteroscopy, myomectomy has one of the highest complication rates. 9

The complications of surgical hysteroscopy can be divided into early and late. 10 Among the early complications, excessive bleeding, uterine perforation, postoperative infection and fluid overload can be mentioned. Some studies 11 12 13 14 report that early complications occur in ∼ 1.0% to 3.6% of the procedures. Regarding the late complications, uterine synechiae are the most frequent. 10 An incomplete resection of a myoma is a suboptimal outcome. A study 15 suggests that in ∼ 12% of the cases it is not possible to perform complete myomectomy in a single procedure, which can result in the exposure of the women to new risk in a subsequent procedure, in addition to resulting in more costs to health systems.

Knowing the factors associated with the complications of hysteroscopic myomectomy is important for an adequate therapeutic planning. In selected cases, the use of medications that decrease the volume of the fibroid prior to surgery, or strategies to facilitate cervical dilatation, may prevent multiple surgical procedures. With the objective of evaluating the factors associated with complete myomectomy in a single surgical procedure, and the aspects related to a higher frequency of early complications of hysteroscopic myomectomy, a retrospective study was conducted in a university hospital.

## Methods

A cross-sectional study was performed, with collection of retrospective data, at Hospital da Mulher Professor Dr. José Aristodemo Pinotti-Centro de Atenção Integrada à Saúde da Mulher, Universidade Estadual de Campinas (CAISM/UNICAMP), including all women who underwent surgical hysteroscopy for the treatment of uterine fibroids from March 1st, 2000 to July 31st, 2017. This hospital is responsible for the training of medical residents, and the surgical procedures are performed by the residents under direct supervision of the senior surgeon. All hysteroscopies were performed in a surgical center adequately equipped to perform the procedure. All procedures were performed under anesthesia, usually spinal block, supplemented or not by drugs with central hypnotic action. The technical steps were performed as recommended by references to perform surgical hysteroscopy. 16 For women who underwent more than one surgical hysteroscopy, only the first procedure was included.

To calculate the sample size, we used the prevalence rate of complications of 2.7%, found in the study by Propst et al. 14 Considering a sample error of 2.5% and a level of significance of 5%, the sample size should be composed of at least 162 women. Through a list provided by the Hospital Statistics Service, 4,826 hysteroscopy procedures were performed between March 1st, 2000 and June 28th, 2016. In the same period, 1,212 hospital admissions with International Classification of Diseases (ICD) corresponding to uterine myoma (D25.0, D25.1, D25.9) were identified. When performing the data crossing, we observed that 227 surgical hysteroscopy procedures were performed to treat myomas.

**Fig. 1:**
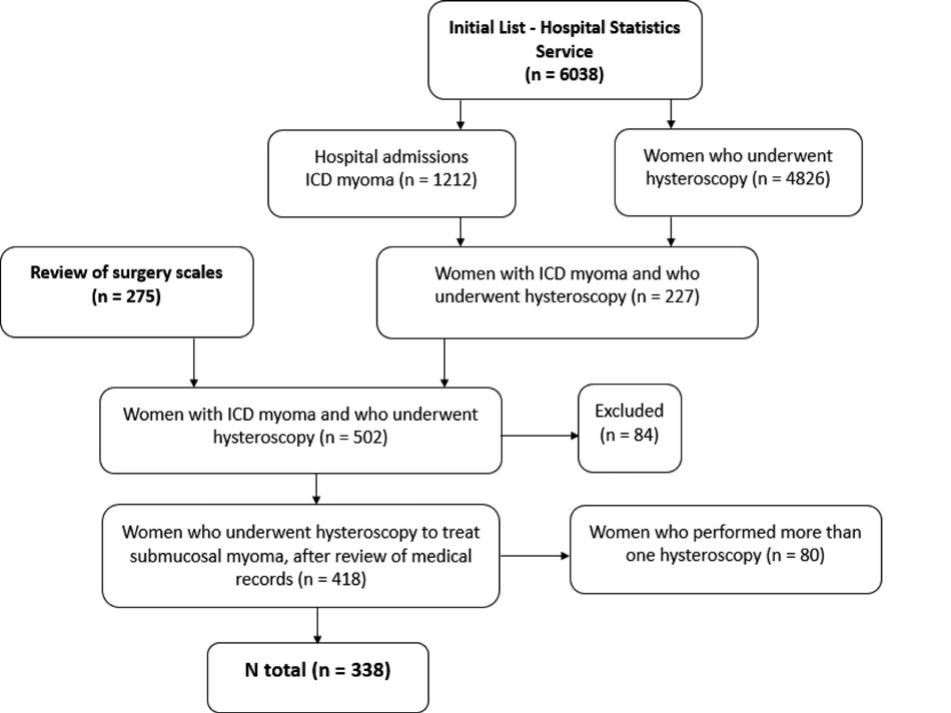
Sample selection.

Later, using the hospital's computerized system, the surgical scales performed between August 1st, 2012 (date of the beginning of the use of the computerized system) and July 31st, 2017 were reviewed, and an additional 275 hysteroscopic procedures that were not present in the initial listing were identified. After a detailed evaluation of the medical records, 84 procedures were excluded from the analysis because they were not surgical hysteroscopies for the treatment of fibroids, making a total of 418 hysteroscopic procedures. For women who underwent more than one procedure, only the first procedure performed at the hospital was included in the analysis. Thus, the final sample consisted of 338 women ( Fig. 1 ).

Data collection was performed by the researcher in charge at the Medical and Statistical Archive Service of the hospital, after careful analysis of the medical records. The project was approved by the Ethics in Research Committee of UNICAMP under CAAE number: 61606216.1.0000.5404.

## Main Outcomes

The main outcomes analyzed in the study were complete myomectomy in a single hysteroscopic procedure and the occurrence of early complications related to the procedure, defined as the presence of any of the following conditions: abnormal bleeding during the procedure; uterine perforation; false route (formation of a different path than the cervical canal during the surgical procedure); fluid overload (occurrence of a deficit in the distension media distention greater than 1000 mL or serum sodium dosage lower than 125 mEq/liter); need for blood transfusion; postoperative infection; and need for laparotomy to treat the complications in the same hospitalization. 17

## Control Variables

The control variables were: longest fibroid diameter in millimeters; fibroid location in the uterine cavity (anterior, posterior, lateral and cervical); International Federation of Gynecology and Obstetrics (FIGO) classification, 18 based on the myometrial component, classified by hysteroscopy or ultrasonography (0/1/2); number of submucous myomas; duration of the procedure in minutes; volume of the distention media used (mL); water balance (mL); indication for the hysteroscopy (abnormal uterine bleeding; infertility; pelvic pain; and abnormal routine-examination finding); result of the anatomopathological examination; age in years; weight (kg); body mass index (BMI); skin color (white and non-white); number of pregnancies; number of vaginal deliveries; number of cesarean sections; number of abortions; menopausal status (premenopausal; postmenopausal); use of hormone therapy to treat menopausal symptoms for at least six consecutive months before surgery (yes/no); use of preoperative gonadotrophin-releasing hormone (GnRH) analog medication for at least three consecutive months prior to surgery (yes/no); use of hormonal combined contraceptive estrogen and progesterone, orally, injected, transdermally or vaginally, for at least six consecutive months before surgery (yes/no); use of progesterone contraceptive alone, orally, injected or subdermally implanted for at least six consecutive months prior to surgery (yes/no); use of misoprostol to prepare the uterine cervix prior to surgery (yes/no); diabetes mellitus (yes/no); arterial hypertension (yes/no); and multimorbidity: presence of two or more chronic diseases (yes/no).

## Statistical Analysis

A descriptive statistical analysis of the data was performed first. The continuous variables were expressed as mean, standard deviation, median, minimum and maximum values. The categorical variables were expressed as relative frequencies. Subsequently, bivariate analyses were performed to verify the association among the dependent variables “complete myomectomy in a single hysteroscopic procedure” and “presence of early complications related to the procedure” and the independent variables. For the categorical independent variables, the chi-squared or Fisher exact tests were performed; the Mann-Whitney test was performed for the continuous variables. Two models of Cox multiple regression were then developed using the Stepwise variable selection criterion to evaluate the factors independently associated with complete myomectomy and early complications related to the procedure. The level of significance was assumed at 5%. The computer software used was The Statistical Analysis System for Windows, version 9.2 (SAS Institute, Inc., Cary, NC, US). 19 20 21 22 23

## Results

In the evaluated period, we identified 338 women who underwent hysteroscopy for the treatment of uterine fibroids. The mean age of the women at the time of the procedure was 47.88 (±11.55) years, and the mean BMI was 28.80 (±5.93). Most women were white (73.43%). The mean number of pregnancies was 2.61 (±2.03), the mean number of vaginal deliveries was 1.46 (±1.89), and the mean number of cesarean deliveries was 0.81 (±1.05). In total, 118 (34.91%) women were postmenopausal, 5.2% of whom were undergoing hormonal therapy to treat climacteric symptoms. A total of 220 (65.09%) women were premenopausal, 70 (20.7%) were using combined hormonal contraceptives, and 56 (16.57%) were using progesterone-only contraceptive methods. Only 10 (2.96%) women had previously used GnRH analogues. The most frequent comorbidities were hypertension 119 (35.31%) and diabetes mellitus 42 (12.43%). In 52 (15.38%) of the procedures, the women had multimorbidity (more than two associated diseases) ( Table 1 ).

**Table 1:** Clinical and sociodemographic characteristics ( *n* = 338)

Characteristics	Total	%
*Age (years)*		
< 40	87	25.74
40–59	192	56.80
> 60–69	59	17.46
Skin color*		
White	246	73.43
Non-white	89	26.57
* Body mass index (kg/m ^2^ ) *		
< 18.5	4	1.18
18.5–24.9	82	24.26
25–29.9	138	40.83
> 30	114	33.73
*Number of pregnancies*		
0	47	13.90
1	59	17.46
≥ 2	232	68.64
*Number of cesarian sections*		
0	180	53.25
1	82	24.26
≥ 2	76	22.49
*Number of vaginal deliveries*		
0	151	44.67
1	61	18.05
≥ 2	126	37.28
*Menopausal status*		
Premenopausal	220	65.09
Postmenopausal	118	34.91
* Menopausal hormone therapy ^a^*		
Yes	6	5.22
No	109	94.78
*Combined hormonal contraceptives*		
Yes	70	20.71
No	268	79.29
*Progesterone-only contraceptives*		
Yes	56	16.57
No	282	83.43
*Gonadotrophin-releasing hormone analogues*		
Yes	10	2.96
No	328	97.04
Hypertension*		
Yes	119	35.31
No	218	64.69
*Diabetes mellitus*		
Yes	42	12.43
No	296	87.57
*Multimorbidity*		
Yes	52	15.38
No	286	84.62

Notes: *Missing data. ^a^ Among postmenopausal women.

Most procedures (88.16%) were performed between 2012 and 2017. The main surgical indication was bleeding in 78.7% of cases, and the second main indication was an incidental finding in a routine imaging exam (19.23%). In 89.05% of the procedures, there was a single fibroid to be treated, in 8.58% of them, there were two fibroids to be treated, and in 2.37%, there were 3 or more fibroids. The mean diameter of the largest fibroid was 2.62 cm (±1.54 cm), the smallest fibroid had 3 mm, and the largest one had 8 cm. According to the International Federation of Gynecology and Obstetrics (Fédération Internationale de Gynécologie et d'Obstétrique, FIGO, in French) most women (66.96%) had fibroids classified as degree 0; 20.54% had grade-1 fibroids, and 12.50% had grade-2 fibroids. The most frequent location of the fibroids was the lateral wall (48.88%), followed by the anterior wall (31.3%), and the posterior wall (28.4%). In 156 (46.15%) of the procedures, misoprostol was used to prepare the uterine cervix. The mean duration of the surgery, after the resectoscope was introduced, was 30.96 minutes (±13.98), with a median of 30 minutes, a minimum time of 3 minutes, and a maximum time of 72 minutes ( Table 2 ).

**Table 2:** Characteristics of the hysteroscopic procedure ( *n* = 338)

Characteristics	Total	%
*Main indication*		
Bleeding	266	78.70
Incidental finding	65	19.23
Infertility	4	1.18
Pain	3	0.89
*Number of fibroids*		
1	301	89.05
2	29	8.58
≥ 3	8	2.37
* FIGO classification ^a,b^*		
0	225	66.96
1	69	20.54
2	42	12.50
* Location ^a,c^*		
Posterior wall	89	25.95
Anterior wall	98	28.57
Lateral wall	153	44.61
Cervical	3	0.87
*Misoprostol*		
Yes	156	46.15
No	182	53.85
*Anatomopathological finding*		
Leiomyoma	286	84.62
Adenomyoma	33	9.76
Lipoleiomyoma	3	0.89
Endometrium	1	0.30
No material	15	4.44

Abbreviation: FIGO, International Federation of Gynecology and Obstetrics (Fédération Internationale de Gynécologie et d'Obstétrique, in French).

Notes: ^a^ Missing data. ^b^ Greatest degree of penetration of the fibroids. ^c^ Location of any fibroid; the women could have more than one fibroid in the uterine cavity.

The myomectomies were completed in 1 procedure in 63.31% of the cases (214 out of 338). In the bivariate analysis of the quantitative variables associated with complete myomectomy in a single hysteroscopic procedure, we found that women at an older age ( *p* < 0.01), with a higher number of pregnancies ( *p* < 0.01), a higher number of vaginal deliveries ( *p* < 0.01), a higher weight ( *p* = 0.04), with less fibroids ( *p* < 0.01), with a lower mean diameter of the largest fibroid ( *p* < 0.01), with shorter surgery duration ( *p* < 0.01), and in which smaller solution volumes were used ( *p* < 0.01) had complete myomectomy with a greater frequency ( Table 3 ). In the bivariate analysis of the categorical variables associated with complete myomectomy in a single hysteroscopic procedure, we found that women with a lower FIGO classification of the largest myoma ( *p* < 0.01), with a non-localized myoma in the anterior wall ( *p* = 0.04), adenomyoma in the anatomopathological examination ( *p* < 0.01), not using combined contraceptives ( *p* < 0.01), no GnRH analogues prior to surgery ( *p* < 0.01), postmenopausal ( *p* < 0.01), with hypertension ( *p* = 0.02), without excessive bleeding during the procedure ( *p* = 0.01), without uterine perforation ( *p* = 0.02), without fluid overload ( *p* < 0.01), and without early complications related to the procedure ( *p* < 0.01) had a greater frequency of complete myomectomies ( Table 4 ).

**Table 3:** Distribution of some quantitative variables according to complete myomectomy and early complications in women undergoing surgical hysteroscopy ( *n* = 338)

	Complete myomectomy - mean (standard deviation)		*p* -value*	Early complications - mean (standard deviation)		*p* -value*
	Yes	No		Yes	No	
Age (years)	49.64 (11.13)	44.83 (11.66)	< 0.01	47.93 (12.44)	47.87 (11.43)	0.72
Pregnancies	2.85 (2.05)	2.19 (1.92)	< 0.01	2.05 (1.84)	2.69 (2.04)	0.02
Vaginal deliveries	1.66 (1.94)	1.10 (1.75)	< 0.01	0.98 (1.73)	1.53 (1.91)	0.02
Weight (kg)	74.12 (14.63)	71.62 (16.87)	0.04	72.16 (15.98)	73.36 (15.47)	0.64
Number of fibroids	1.09 (0.34)	1.24 (0.63)	< 0.01	1.20 (0.51)	1.14 (0.47)	0.25
Mean diameter of the largest fibroid (mm)	21.66 (12.29)	36.15 (15.84)	< 0.01	32.02 (15.78)	26.22 (15.18)	0.02
Duration of the procedure (minutes)	29.36 (13.16)	33.73 (14.95)	< 0.01	33.00 (18.18)	30.66 (13.25)	0.55
Volume of the distension media (ml)	102.80 (1024.90)	689.52 (2587.20)	< 0.01	647.73 (2591.50)	268.71 (1631.4)	0.20

Note: *Mann-Whitney test.

**Table 4:** Categorical variables associated with complete myomectomy and early complications in women undergoing surgical hysteroscopy ( *n* = 338)

	Complete myomectomy (%)		n	*p* -value	Early complication (%)		n	*p* -value
	Yes	No			Yes	No		
FIGO classification of the largest fibroid*				< 0.01 ^a^				0.96 ^a^
0	71.56	28.44	225		13.33	86.67	225	
1	53.62	46.38	69		13.04	86.96	69	
2	33.33	66.67	42		88.10	11.90	42	
Anterior-wall fibroid*				0.04 ^a^				0.62 ^a^
Yes	56.12	43.88	98		10.20	89.80	98	
No	67.90	32.10	215		12.09	87.91	215	
*Anatomopathological finding*				< 0.01 ^b^				0.40 ^b^
Leiomyoma	63.63	36.37	286		11.19	88.81	286	
Adenomyoma	93.94	6.06	33		9.09	90.91	33	
Other	5.26	94.74	19		47.37	52.63	19	
*Combined hormonal contraceptives*				< 0.01 ^a^				0.96 ^a^
Yes	47.14	52.86	70		12.86	87.14	70	
No	67.54	32.46	268		13.06	86.94	268	
*Misoprostol*				0.33 ^a^				0.23 ^a^
Yes	66.02	33.98	156		15.38	84.62	156	
No	60.99	39.01	182		10.99	89.01	182	
*Progesterone-only contraceptives*				0.17 ^a^				0.04 ^a^
Yes	55.36	44.64	56		21.43	78.57	56	
No	64.90	35.10	282		11.35	88.65	282	
*Gonadotrophin-releasing hormone* *analogue*				< 0.01 ^b^				0.62 ^b^
Yes	0.00	100.00	10		20.00	80.00	10	
No	65.24	34.76	328		12.80	87.20	328	
*Menopausal status*				< 0.01 ^a^				0.37 ^a^
Postmenopausal	75.42	24.58	118		15.25	84.75	118	
Premenopausal	56.82	43.18	220		11.82	88.18	220	
*Hypertension*				0.02 ^a^				0.40 ^a^
Yes	71.43	28.57	119		15.13	84.87	119	
No	59.17	40.83	218		11.93	88.07	218	
*Excessive bleeding*				0.01 ^a^				–
Yes	33.33	66.67	15		–	–	–	
No	64.71	35.29	323		–	–	–	
*Uterine perforation*				0.02 ^a^				–
Yes	35.71	64.29	14		–	–	–	
No	64.51	35.49	324		–	–	–	
*Fluid overload*				< 0.01 ^b^				–
Yes	0.00	100.00	6		–	–	–	
No	64.46	35.54	332		–	–	–	
*Early complication*				< 0.01 ^a^				–
Yes	34.09	65.91	44		–	–	–	
No	67.69	32.31	294		–	–	–	
*Complete myomectomy*				–				< 0.01 ^a^
Yes	–	–	–		7.01	92.99	214	
No	–	–	–		23.39	76.61	124	

Abbreviation: FIGO, International Federation of Gynecology and Obstetrics (Fédération Internationale de Gynécologie et d'Obstétrique, in French).

Notes: ^a^ Chi-square test. ^b^ Fisher exact test. *Missing data.

Throughout the analyzed period, 44 (13.01%) women had early complications related to the hysteroscopic procedure: 15 (4.44%) presented excessive bleeding during the procedure, 14 (4.14%) had uterine perforation, 9 (2.66%) had a false route, 6 (1.78%) presented fluid overload, 2 (0.59%) underwent laparotomy, and 1 (0.3%) had a postoperative infection. No woman needed blood transfusion after the procedure. In the bivariate analysis of the variables associated with early complications, we found that women with fewer pregnancies ( *p* = 0.02), a lower number of vaginal deliveries ( *p* = 0.02), fibroids with longer diameters ( *p* = 0.04), with incomplete myomectomy ( *p* < 0.01), and who used progesterone-only contraceptives ( *p* = 0.04) had early complications with a higher frequency ( Tables 3 and 4 ).

In the final statistical model using the multiple Cox regression, we observed that the factors independently associated with complete myomectomy in a single hysteroscopic procedure were the diameter of the largest fibroid in millimeters (PR: 0.97; 95%CI: 0.96–0.98) and fibroid FIGO classification 0 (PR: 2.04; 95%CI: 1.18–3.52). The only independent factor associated with the occurrence of early complications was incomplete myomectomy (PR: 2.77; 95%CI: 1.43–5.38) ( Table 5 ).

**Table 5:** Variables associated with complete myomectomy and early complications – Multiple Cox Regression ( *n* = 313)

	Categories	*p* -value	PR	95%CI (PR)
**Complete myomectomy** a Largest fibroid diameter	Continuous variable (mm)	< 0.01	0.97	0.96–0.98
FIGO classification	2 (ref)	—	1.00	—
	1	0.10	1.68	0.91–3.10
	0	0.01	2.04	1.18–3.52
**Early complications** b				
Complete myomectomy	Yes (ref)	—	1.00	—
	No	< 0.01	2.77	1.43–5.38

Abbreviation: 95%CI, 95% confidence interval; FIGO, International Federation of Gynecology and Obstetrics (Fédération Internationale de Gynécologie et d'Obstétrique, in French); PR, prevalence ratio; ref, reference level.

a PR, prevalence ratio for complete myomectomy ( *n* = 105 no; *n* = 208 yes).

b PR, prevalence ratio for early complications ( *n* = 277 no; *n* = 36 yes); 95%CI PR = 95% confidence interval for the prevalence ratio. Stepwise criteria for variable selection.

## Discussion

Submucosal myomas represent 5% to 10% of all myomas. 24 Hysteroscopic myomectomy is the treatment of choice, but among the hysteroscopic procedures, it is the one with the highest complication rates. 14 Our objective was to identify the factors related to the possibility of complete myomectomy in a single surgical procedure, in addition to identifying the possible factors associated with a higher frequency of early complications resulting from the procedure.

In the present study, we found a complete myomectomy rate of 63.31%, which is lower than the one reported by Mazzon et al 15 in 2015, which was of 87.62%. However, in their study, Mazzon et al 15 included only women with single fibroids. In the present study, ∼ 10% of the women had more than one submucosal fibroid to be treated, which may justify the difference observed. In 2008, Murakami et al 25 reported a complete myomectomy rate of 57.1%; however, the authors included only women with fibroids of degrees 1 and 2 according to the FIGO classification. These fibroids are technically more difficult to be resected in comparison to pediculate fibroids (grade 0), which impairs the comparison between the studies.

We observed that the factors independently associated with complete myomectomy in a single surgical time were the fibroid diameter and the degree of penetration in the myometrium. With each 1-mm increase in myoma diameter, the chance of complete myomectomy decreased by 2.4%; in addition, women with fibroids classified as FIGO grade 0 had twice the chance of having a complete myomectomy compared with women with FIGO grade-2 fibroids. Our data are in agreement with that of other studies on the subject. Wamsteker et al 18 conducted a prospective study with 51 women with submucosal fibroids and increased bleeding, and they concluded that myomas with greater myometrial penetration would have less chance of complete resection, requiring multiple procedures to improve the symptoms. In addition, fibroids with a larger intramural component generally have a larger diameter and volume, which increases the complexity of the procedure, reducing the probability of complete myomectomy in a single surgical time. 6

We did not observe an association between the previous use of GnRH analogues and a greater frequency of complete myomectomies. In contrast, no woman who had previously used GnRH analogues had a complete myomectomy in a single surgical time. This finding is probably due to the fact that even after using GnRH analogues, the women had fibroids larger than the general mean (the mean overall diameter was 2.6 cm, and the mean diameter in women who had used GnRH analogues was 4.9 cm). Similarly, in the randomized controlled trial by Favilli et al, 26 prior use of GnRH analogues did not facilitate the complete removal of grade-2 submucosal fibroids and increased the surgical time. In addition, we did not find any independent association between contraceptive use (combined or progesterone-only) and complete myomectomy or early complications.

In the present study, early complications were observed in 13.02% of the procedures, a rate similar to that found by Propst et al, 14 who reported a 14% complication rate in hysteroscopic myomectomies. Of all early complications we observed, the majority (4.44% of the cases) was excessive bleeding during the procedure. The classification of excessive bleeding was based on the reports described in the medical records, which are subject to bias. The quantification of the bleeding volume in the procedure is difficult to perform accurately. A fact that corroborates this finding is that, despite the report of excessive bleeding in the description of the surgery, no women required blood transfusion. Not all women had their hemoglobin levels measured before and after the procedure, which made it impossible to use this parameter in the analysis. The second and third most frequent complications were uterine perforation (4.14%) and false route (2.66%). Of the uterine perforations, 6 were at dilation of the uterine cervix. In a randomized controlled trial, Preutthipan and Herabutya 27 concluded that the use of vaginal misoprostol may prevent the risk of uterine perforation. However, in our study, the use of misoprostol was not associated with fewer complications, and was not associated with the complete resection of fibroids. A recent clinical trial 28 also evaluated the use of misoprostol as a method of cervical preparation, but prior to the performance of the diagnostic hysteroscopy in postmenopausal women. The authors concluded that in addition to misoprostol not reducing the intensity of the pain, the duration of the procedure and the need for additional cervical dilatation, there was a higher incidence of adverse events, such as vaginal bleeding, cramps and diarrhea among medication users. 28

Fluid overload can occur through direct passage of fluid through the uterine vessels that are opened during surgery or through the entry of fluid into the peritoneal cavity, either through the uterine tubes or through uterine perforation. 29 In the present study, the only distension medium used was 1.5% glycine, and 6 women presented with fluid overload (1.78%). Among them, none had uterine perforation. In a Dutch multicenter study, 13 the authors identified 5 cases of fluid overload in 2,515 surgical hysteroscopies (0.2%), although, in that study, only 35% of the health facilities were teaching hospitals, which could partly explain the difference in fluid overload rates.

The present study has some limitations. Since it is a cross-sectional study, it is not possible to establish cause and effect associations. We could not use the size, topography, extension, penetration, wall (STEPW) classification proposed by Lasmar et al, 30 31 because we did not have access to data regarding the extent of the myoma base in relation to the uterine wall. The classification adopted by the FIGO was used to classify the myometrial component, which is currently more widespread and used than the STEPW classification. 18 Although we did not use the STEPW classification, we did not observe an association between the location of the myoma in the uterine cavity and a greater frequency of incomplete myomectomies or early complications. The fact that the only independent factor associated with the occurrence of early complications was incomplete myomectomy is probably an interpretation bias. Since the diameter of the fibroid and the degree of penetration in the myometrium are independently associated with a complete myomectomy, they are also probably associated with the occurrence of early complications. The trend of evolution and dissemination of minimally-invasive technologies contributed to the increase in the number of hysteroscopies performed during the analyzed period; however, since 2012 the surgical information control was completely computerized, which may have contributed to an easier identification of women undergoing hysteroscopic myomectomy starting that year.

## Conclusion

In conclusion, our results corroborate the fact that the main factor associated with complications in the hysteroscopic myomectomy procedure is the myoma itself. No other characteristic, whether intrinsic to the women, such as age, skin color, parity and presence of comorbidities, or even treatment-related characteristics, such as use of GnRH analogues, misoprostol or hormonal contraceptives, was associated with a higher frequency of incomplete myomectomies or early complications. Women with larger fibroids and with a high degree of myometrial penetration have a greater chance of developing complications of hysteroscopic myomectomy.
